# An Immersive Human-Robot Interactive Game Framework Based on Deep Learning for Children’s Concentration Training

**DOI:** 10.3390/healthcare10091779

**Published:** 2022-09-15

**Authors:** Li Liu, Yangguang Liu, Xiao-Zhi Gao, Xiaomin Zhang

**Affiliations:** 1 College of Digital Technology and Engineering, Ningbo University of Finance and Economics, Ningbo 315175, China; 2 College of Finance and Information, Ningbo University of Finance and Economics, Ningbo 315175, China; 3 School of Computing, University of Eastern Finland, 70210 Kuopio, Finland

**Keywords:** concentration training, human–robot interaction, gesture recognition, deep learning

## Abstract

In order to alleviate bottlenecks such as the lack of professional teachers, inattention during training processes, and low effectiveness in concentration training, we have proposed an immersive human–robot interactive (HRI) game framework based on deep learning for children’s concentration training and demonstrated its use through human–robot interactive games based on gesture recognition. The HRI game framework includes four functional modules: video data acquisition, image recognition modeling, a deep learning algorithm (YOLOv5), and information feedback. First, we built a gesture recognition model containing 10,000 pictures of children’s gestures, using the YOLOv5 algorithm. The average accuracy in recognition trainingwas 98.7%. Second, we recruited 120 children with attention deficits (aged from 9 to 12 years) to play the HRI games, including 60 girls and 60 boys. In the HRI game experiment, we obtained 8640 sample data, which were normalized and processed.According to the results, we found that the girls had better visual short-term memory and a shorter response time than boys. The research results showed that HRI games had a high efficacy, convenience, and full freedom, making them appropriate for children’s concentration training.

## 1. Introduction

Attention is an important indicator of a child’s physical and mental health. Attention deficit hyperactivity disorder (ADHD) is one of the most common behavioral problems in childhood, with an incidence of 4.31 to 5.83 percent of school-aged children in China, and the peak age of the disease is mostly concentrated at ages eight and nine, with a male-to-female ratio of 9:1 [1]. Children with ADHD often exhibit behavioral traits such as an inability to concentrate, overactivity, and impulsivity, which cause them to encounter different degrees of difficulty in school and life [2]. At the same time, the symptoms of ADHD can lead to a range of psychological problems, including low self-esteem, peer rejection, low self-confidence, and poor adaptability [3]. Multiple studies have shown that 30 to 50 percent of children with ADHD have symptoms that do not disappear naturally with age [4], due to the chronic nature of ADHD symptoms. Effective intervention training is needed for children with ADHD. In recent years, researchers have divided the attention training of children with ADHD into three categories: neurophysiological training, pen-and-paper training, and behavioral training.

Neurophysiological training includes two main methods: brain wave stimulation and drug therapy. Stimulant medication can quickly relieve ADHD symptoms but has side effects such as decreased appetite, difficulty falling asleep, and headaches [5]. At the same time, medication does not improve the mental health of children with ADHD, and many children with ADHD have persistent residual symptoms after receiving medication treatment. Thus, neurophysiological training mainly manipulates concentration from a physiological point of view, which entails certain risks [6]. Therefore, it is necessary to explore new interventions to help children with ADHD improve their symptoms and better adapt to society.

Pen-and-paper training mainly includes assessment scale training and pen-and-paper game training, which is a traditional attention training method that is time-consuming and labor-intensive. Parents or teachers are trained to fill in the Conners behavior scale [7], which includes six items of character problems, learning problems, psychosomatic disorders, impulse hyperactivity, anxiety, and the hyperactivity index to understand aspects of children’s attention. Pen-and-paper tests mainly include scratch tests, digital breadth tests, writing tests, symbolic number pattern tests, etc. [8]. Mindful concentration training is an intervention program for adolescent trait anxiety and social avoidance, in which the effectiveness of the intervention is verified through various scale tests [9]. Cognitive training games have improved attention and executive function in school-aged children with ADHD through an adaptive repetitive hierarchical grading exercise [10]. The one-to-one approach uses progressive level-breaking games to train concentration, assessing whether the intervention is sustainable based on the results of participants’ play performance and behavioral responses. The pen-and-paper training method is used to evaluate indicators such as persistence, selectivity, attention, inhibitory control, and working memory through experiments, which require a large number of teachers or parents and children to conduct one-on-one training. Therefore, this approach is time-consuming and inefficient.

Behavioral training is a potential method that combines traditional attention training methods with computer technology to reduce human interference in the training process, such as reducing scoring errors and improving efficiency. The most common method of attention training for children is the Stroop Word Color Interference Test (SWIT) [11]. The Attention Continuity Performance Test (CPT) has been developed to test the scope of children’s persistent attention. Visual task training includes five training modules—instruction reception training, gaze training, visual tracking training, visual search training, and visual memory training [12]. The principle of concentration training based on vision games involves the use of situational dialogue and emotional expression in one-on-one or many-to-one modesto help children with ADHD improve their attentiveness. At present, although some reviews have been conducted on human attention training based on human–computer interaction, there is no guidance for robot design in this field [13,14,15,16,17]. A prototype design of a YOLO robot has been proposed, which could serve as a tool to stimulate new ideas and stimulate creativity, providing educational strategies for children’s creative development [13]. Safinah Ali et al. designed a social robot for children based on the interactive mode of artificial creativity and experimentally demonstrated that children’s creativity was improved in the interactive game with the social robot [14]. The findings of a survey of social robots indicated that social robots can be used as assistive robots for children’s play, learning, and cognitive development. A computer-aided technique for the testing of ADHD patients was proposed, which has gained FDA approval for the objective measurement of hyperactivity, impulsivity, and inattention, and for the assessment of ADHD [15]. One of the major challenges in the use of human–robot interaction in concentration training is to improve the random freedom of HRI. For example, humans and robots are free and open in games, not restricted by third parties, and real-time interactivity has become an important way of improving the effects of HRI games [18]. Therefore, the question of how to improve the real-time aspect and the degree of freedom in HRI games needs to be further explored. Immersive games offer a possible alternative. An immersive learning mode, combined with the use of a robot and virtual reality technology, played a positive role in improving efficiency in English learning and effectively promoting the balanced development of education [15]. However, this method involves high requirements in terms of equipment, as well as a high cost and low efficiency.

According to the above findings, concentration training needs to solve the problems of the shortage of teachers, low efficiency, and high cost. With the development of human–robot interaction technology, HRI games have become a popular trend for children’s concentration training. However, non-verbal communication is very important in human–robot interaction just as it is in human–human interaction [19,20,21]. Thus, natural interaction and real-time performance are two key issues in relation to the application of HRI games in concentration training. Experiments have shown that this new HRI game scenario is beneficial for concentration training, and there are thus two questions that need to be explored regarding natural relationships and reaction time [22,23,24,25]:

1. Does the gesture recognition-based immersive HRI game scenario realize the goal of unconstrained concentration training?
2. Are the contents of the immersive HRI game suitable for children’s concentration training, such as for visual short-time memory and response time?

The remainder of this paper is organized as follows. The second section discusses the related work. The third section presents the materials and methods. The findings from the content analysis of the related papers are described in the fourth section. Finally, the fifth section concludes and provides suggestions for further developments.

## 2. Related Work

In this section, we briefly introduce gesture recognition based on deep learning and human–robot interactive games.

### 2.1. Gesture Recognition Based on Deep Learning

Gesture recognition is a growing field of computer science. Gesture recognition computer processes are designed to enhance human–robot interaction and can occur in multiple ways, such as using touch screens, cameras, or peripheral devices. In human–robot interaction, high-precision and real-time gesture detection and recognition are the most basic preconditions for acquiring hand information. Dynamic gesture recognition based on real-time video is one of the most popular research fields in computer vision, and the core of dynamic gesture recognition is a target detection algorithm based on deep learning.

**Figure 1:**
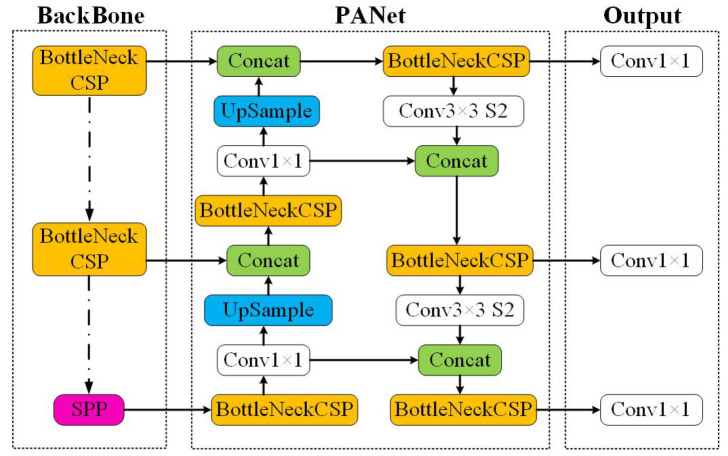
YOLOv5 model architecture.

The object detection algorithm based on deep learning is mainly divided into two types: (1) the two-stage algorithm represented by the R-CNN series and (2) the one-stage algorithm represented by YOLO [22] and SSD [23]. Specifically, the two-stage algorithm first generates candidate regions on the image and then classifies and returns the boundaries of each candidate region in turn. The one-stage algorithm directly locates and classifies all targets on the entire image, skipping the step of generating candidate regions. The one-stage detection network performs classification and bounding box regression at the same time as the candidate box is produced, and this method is characterized by a high speed but slightly less precision. YOLOv1 was introduced in 2016 by Joseph Redmon et al. [26], then YOLOv3 [27] was introduced to improve upon YOLOv2 [28]. Shortly after the release of YOLOv4 [20], Glenn Jocher introduced YOLOv5 using the Pytorch framework, which splits images into S × S meshes and directly predicts category probability and regression position information based on the bounding box corresponding to each mesh. The YOLOv5 algorithm was optimized on a convolutional neural network (CNN), changing the number of connection layers and connection methods of convolutional and pooled layers. Training the gesture recognition model on the COCO dataset improved the gesture recognition model, reduced the gradient of the model, and provided higher recognition speed and recognition accuracy. YOLOv5 is the latest and the most lightweight version of the previous YOLO algorithms and uses the PyTorch framework instead of the Darknet framework. An overview of YOLOv5 is shown in Figure 1 [24], and Table 1 summarizes the comparison between the architectures of the YOLOv3, YOLOv4, and YOLOv5 algorithms [29]. The head and neural network type are the same for all of the algorithms, whereas the backbone, neck, and loss function are different.

**Figure 2:**
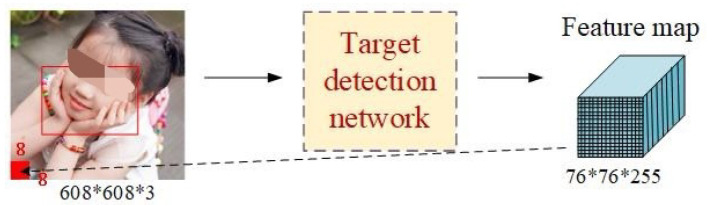
Flowchart of the training process of the YOLO algorithm.

Compared with the current mature large- and medium-scale target detection technologies, the effect of small-scale target detection is relatively poor. Thus, the question of how to improve the detection accuracy of small targets is a difficult one in the field of computer vision. Taking the input 608 × 608 of the network as an example, downsampling in YOLOv3, YOLOv4, and YOLOv5 was carried out five times; so, the final feature map sizes were 19 × 19, 38 × 38, and 76 × 76, as shown in Figure 2. In the three feature maps, the 76 × 76 feature mapwas responsible for detecting small targets and this corresponded to 608 × 608; the receptive field of each feature map was 608/76 = 8 × 8 size. Therefore, YOLOv5’s object detection was more accurate, at more than 97% [22].

Considering the importance of speed and the provision of real-time results in attention training, we used the YOLOv5 algorithm based on deep learning to design an immersive HRI concentration training game.

### 2.2. Human–Robot Interactive Game

The technology of human–robot interaction has gradually been applied to the field of education [23,24,25,30,31]. Logan found that children were more attracted to electronic products and could concentrate and maintain their attention for longer [25]. Clarke acquired measurements of the gestural movements of children when playing tablet games, and assessed whether children had ADHD by analyzing the characteristic vectors of these gestures, obtaining results with up to 90% accuracy [31]. Baer used machine learning algorithms to analyze home videos of 80 children and applied eight machine learning models to evaluate whether children had ADHD [32]. Arns had social robots interact with children in a prescribed scene, while using depth cameras and computer vision technology to analyze children’s head posture, eye gaze range, and direction to achieve an assessment of their common attention [33]. Logan used immersive virtual reality technology to intervene in the emotional recognition ability of children with ADHD, designed multiple virtual scenes of social interaction, and automatically judged the emotional states of children through the use of a computer vision system [25].

### 2.3. The Core Indicators for Evaluating Concentration

The five core indicators for evaluating the quality of attention are concentration, persistence, transference, stability, and breadth [24,25,26,27,28,29,30,31,34]. Focused attention refers to the ability of the brain to pay attention to a specific goal at any time, which means to focus on a specific task that lasts for a period of time without distraction. Persistence is the ability to maintain or focus on something for an extended period of time, even when doing repetitive tasks and activities [25]. Transferability refers to the ability to flexibly shift the focus of attention, enabling a person to work between different cognitive tasks [30]. Stability refers to the ability to maintain attention on a specific object and is the product of duration and quality of focus. Span refers to the number of objects that a person can clearly perceive or recognize at the same time, and is also called attention span. Attention span is related to learning efficiency. In this study, we focused on attention and transferability.

In this paper, we propose a human–robot interaction game based on the use of a deep learning algorithm, which can be applied to children’s concentration training. The effectiveness of this method for children’s attention training was verified by designing gesture recognition games to test hand–eye coordination, visual short-term memory, reaction time, and other indicators that reflect attention and transferability.

## 3. Materials and Methods

At present, common problems in concentration training are related to the fact that the venues and training tools have certain requirements, whereas the game content is singular, fixed, and conventional, and that the activity training process has low accuracy, complexity, and is not conducted in real time [34]. To solve the above problems, an immersive HRI game based on the YOLOv5 algorithm for children’s concentration training was proposed and experimentally verified.

**Figure 3:**
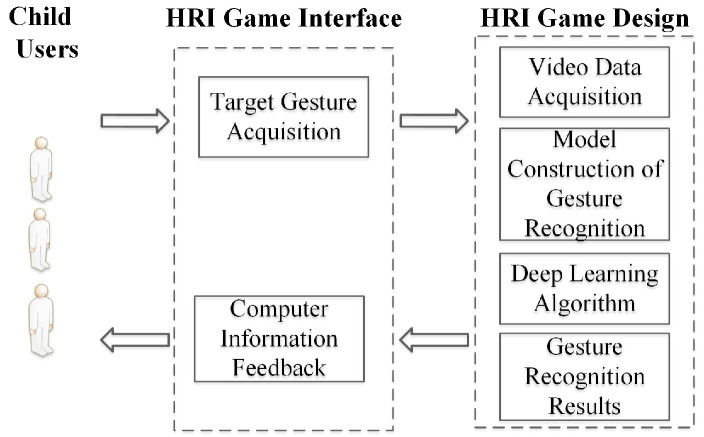
The framework of concentration training method based on human-robot interactive games.

The immersive human–robot interaction game system proposed in this paper includes four functional modules: video data acquisition, model construction of the gesture recognition, a deep learning algorithm, and information feedback, as shown in Figure 3. We designed an immersive human–robot interaction game called “Running Train”, which involved 120 children with attention deficits completing the game. The entire game was videotaped, and we analyzed the indicators of concentration in the experimental results. The game called “Train Run” is a kind of game that Chinese children are generally familiar with, which involves completing a game in sequence. In our experiments, child subjects played games in groups. Four children were included in each group, and they took turns to complete the HRI game according to the game’s rules.

The method of using the proposed immersive HRI game in children’s concentration training has the following advantages. Firstly, the trainee freely completes the concentration training and evaluation during the immersive games with the robot. Secondly, multiple trainees can be trained at the same time, without professional teachers, greatly improving the training efficiency and reducing labor costs. Finally, there was no specific requirements regarding the training location, which is convenient for the market application and promotion of the method.

The concentration training scenarios began with an immersive human–robot interactive game. The user provided feedback through gestures, according to the computer’s random orders, which were recorded in real time by the camera. Then the gesture recognition module processed the recorded gesture data. The functional module contained the definition of gestures, the construction of a gesture training model, the deep learning algorithm, and the feedback of the gesture recognition results.

During the game, four children sat on four fixed chairs and performed corresponding gestures according to the computer’s orders. The children’s gestures were captured by the HRI game system with a webcam. Then, the corresponding gestures were identified. Next, the system provided corresponding feedback to the children. Children played freely without human interference during the HRI games. Every child played with the robot in a real and unobstructed interactive game. During the games, the robot calculated each child’s reaction time and accuracy during the game. Finally, the HRI game produced each sub-result of the game as an output. The experimental setup, procedure, measurement, results, and participant information are described below.

### 3.1. Experimental Setup

**Figure 4:**
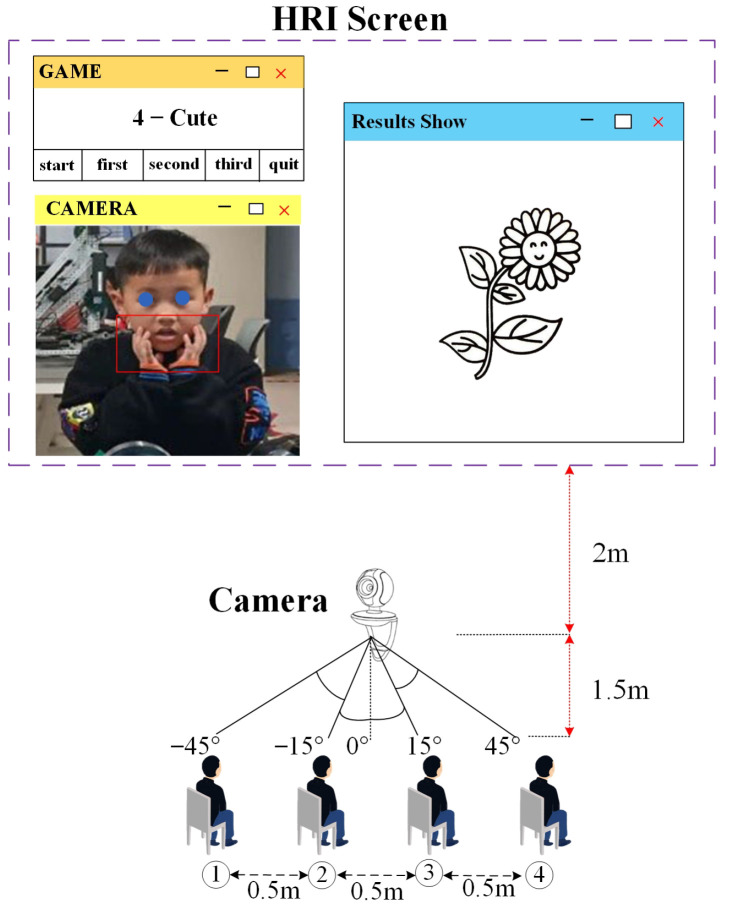
Experimental setup of immersive concentration training games with robots.

The experimental setup contained child users, a camera, wireless computers, and projectors, as shown in Figure 4. To show the process of the children’s game and the game results more specifically, we depict an instance of the children’s game as an example in Figure 4. The experimental site was in an ordinary classroom, and the camera was placed on the podium, two meters away from the large screen. Four chairs were numbered from 1 to 4 and were placed 1.5 m away from the camera in the vertical direction. During the game, the children faced and gestured towards the camera, and directions were projectedonto the wall in the HRI games. That is, during the entire game process, the children faced the wall to play the games. The size of the game screen was about 2.5 m wide and 1.5 m high.The size of the game screen was approximately 2.5 m wide and 1.5 m high. In the layout of the test site, we put the camera above the center of the screen, assuming that the front angle of the camera was 0°. The chair and camera orientations were set to −45°, −15°, 0°, 15°, and 45° from left to right, respectively. Because of limited experimental funding, every child was not given a screen. Four children did not play at the same time. The game was played in groups. Each group of game members included four children, who completed three rounds of games with different requirements. At the same time, only one child completed the gesture recognition game. A specific description of the game instructions is presented in Section 3.2. The purpose of the experiment was to verify whether the human–robot interaction system was effective for children’s concentration training and evaluation.

The immersive human–robot interaction games described here have no venue requirements, and experiments can be carried out freely in ordinary teaching places. No third-party intervention is required during the play process, and concentration training and assessment can be carried out with only children participating in the game. The immersive human–robot interaction concentration training game we designed enables free training at any time, anywhere, and is independent of the conditions of the training site, and thus has a very wide range of applications, such as humanoid robot design for HRI, screening and intervention for children with depression, screening and intervention for children with autism, and so on.

In order to test the concentration training effects of this human–robot interaction game, we selected 120 children with inattention issues from five primary schools in Jiangbei District and Yinzhou, Ningbo City, to conduct a game experiment (age: 9–12 years old, 60 boys, 60 girls). All of these children were able to perform normal imitation behaviors according to the testers, and they had no difficulty in movement; hence, they were acceptable for the study.

### 3.2. Experimental Procedure and Measurement

In the experiment, all child players were divided into groups of four children, assuming A, B, C, and D, and they participated in the gesture game together, with the children being able to play freely and autonomously. Gesture commands were specified by the robot and displayed on a large screen via a projector. Children made corresponding gestures according to the gesture instructions within the limited time of two seconds and were awarded one point for correct completion of the gesture and zero points for incorrect completion. At the same time, the robot recorded the time when the child completed the gesture through the camera. Each group of children played three rounds of games. In the first round of the game, four children in a permutation and combination completed the specified gestures in sites 1 to 4, respectively. In the second round of the game, the four children sat in a fixed site, numbered from 1 to 4, and randomly completed a random gesture, choosing one of the five. The positions and gestures of the four children in the third round were randomly determined. The total number of times each child completed the game in each round was 24, and each child completed three rounds. Since 120 children participated in the experiment, a total of 8640 game results were obtained. We normalized these 8640 data for analysis.

There was no third-party participation in the game process, the game process is videotaped, and the game system provided feedback on the correct or wrong results in real time, according to the gestures made by the children, recording the accumulated game scores of each child. At the same time, the game system also recorded the time at which the children completed the gestures and calculated each child’s reaction time. Finally, we evaluated the effect of the concentration training game by calculating and comparing the accuracy of playtime and movements in all samples.

Concentration assessment indicators included object recognition, visual short-term memory [35], focused attention, response time [36], and so on. The experimental results were processed and analyzed using Python and SPSS software. For the accuracy analysis of the gesture response, we used mixed-effects repeated measures of ANOVA, in which gestures, direction, and order of play were random effects and the physical distance was a fixed effect. During the game, the children underwent concentration assessment and training based on the feedback provided by the machine.

## 4. Results

### 4.1. Gesture Recognition

**Figure 5:**
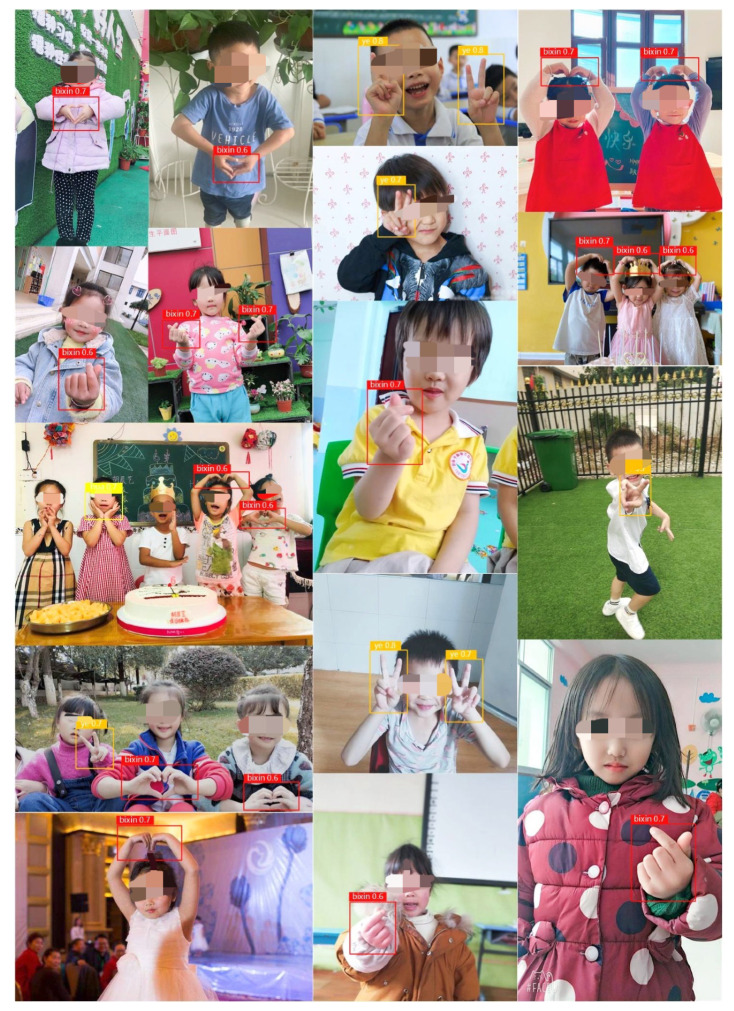
Gesture recognition training samples used for YOLOv5.

In the experiments, we adopted HRI games based on gesture recognition. The gesture recognition algorithm consisted of network training and gesture recognition [37]. In the network training stage, the improved feature extraction network was used to fit the feature distribution of training samples, and the fitting error was estimated according to the deviation between the gesture label and the network output; that is, the recognition result and the updating direction of network weights at all levels were determined by calculating the error gradient. After network training, in the gesture recognition step we used the weight files obtained in the training stage to restore the deep network and then used the network to complete the feature extraction from the test data. Finally, the YOLOv5 target recognition algorithm for this experiment was used to decode the network output data, and the results of the gesture recognition process were obtained. The gesture recognition training samples based on the YOLOv5 algorithm are shown in Figure 5.

The training of the YOLOv5 model was as follows.

Preparing the image dataset. We collected 10,000 images, 2000 images for each of the five gestures that expressed ‘OK’, ‘No’, ‘Yes’, ‘Cute’, and ‘Love’. All images were obtained from partner schools. The dataset contained images in indoor and outdoor environments, images of different gestures by a single person, and different gestures by multiple people in one image. If the images contained different gestures, the images belonged to different gesture classes.

Labeling the image: The labels of the 5 categories were set to 1, 2, 3, 4, and 5, respectively. we used the Colabeler (http://www.colabeler.com/, 17 April 2021) tool to annotate and label our images and exported them in YOLOv5’s annotation format. We used rectangular boxes to label various gestures.

Training the model: We ran a deep learning experiment on a Linux Server with a GPU (NVIDIA GeForce RTX 2080 Ti graphics card). yolov5s.pt was used for the pretrained weighted models in the YOLOv5 algorithm. The number of iterations was set to 3000 batches. The size of each batch was set to 16. The image resolution, as the feature parameter (-imgsz), was set to 640 × 640, and the patience was set to 1000. Other parameters retained their default values.

Evaluating the model: We conducted 5-fold cross-validation in order to evaluate the model’s performance. The average accuracy of the training recognition was 98.7% in the test set.

After we had trained and evaluated the model, we used the model to classify the images from the video that was processed frame by frame and made a prediction for each frame.

### 4.2. Visual Short-Term Memory

**Figure 6:**
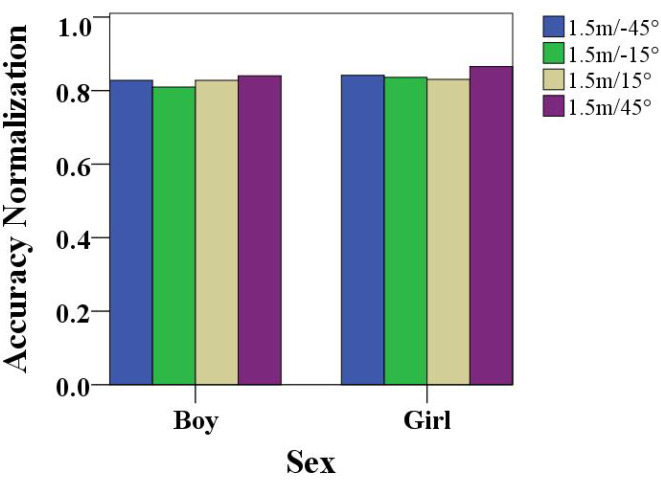
Comparison of the performance of boys and girls playing at different angles.

**Figure 7:**
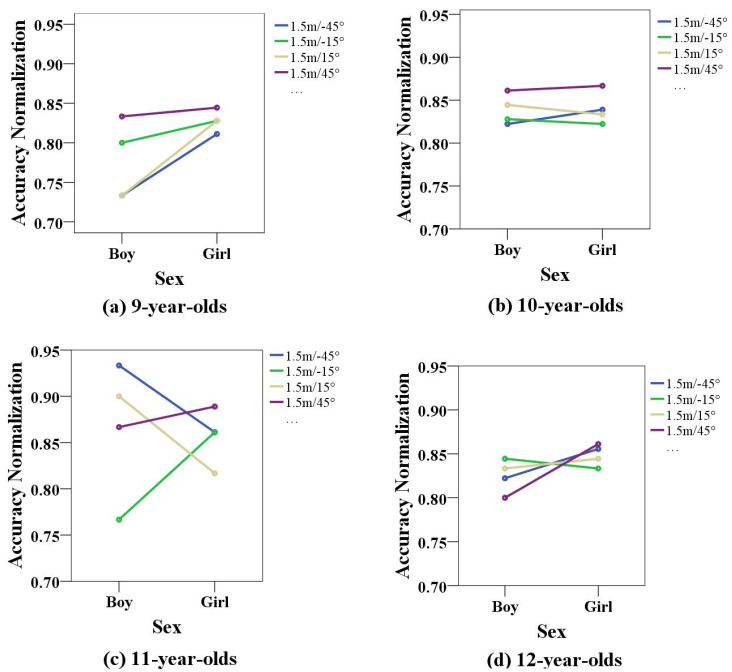
Comparison of children’s play performance at different angles for children of different ages.

The children’s performance in the game was summarized and analyzed. Visual short-term memory is an important characteristic of human concentration. In our experiments, visual short-term memory was measured based on the accuracy value expressed by the gesture completion rate in the games. The higher the accuracy rate, the better the visual short-term memory; therefore, the better the concentration. To facilitate the analysis, the accuracy results were all normalized. There were some factors that affected accuracy, such as location, sex, and age. The effect of these factors on the training effectiveness of the concentration game was analyzed. We specifically discussed the impact of physical distance and orientation on gesture recognition accuracy in our previously published paper [37]. In order to compare and analyze the data, we performed Z-Score normalization on the children’s game scores, which are shown in Figure 6 and Figure 7.

Children sat in fixed chairs, which were placed at the same distances from the camera. A comparison of the performances of boys and girls playing at different angles is shown in Figure 6. When the boys and girls sat at different angles in the chair to complete the gesture game, in terms of the performance standardization value, we observed that the boys’ performances in the −15° angle were low, and the −45°, 15°, and 45° angle scores were relatively high. In particular, boys were more focused at the 45° angle in the attention training game. The 45° angle had the highest performance standard value for girls as well, indicating that the 45° angle was the best angle for children to play attention training games. Meanwhile, girls performed better than boys according to the analysis.

The results of the human–computer interactive games for children of different ages were analyzed, in terms of sex and angle, for children from 9 to 12 years old, as shown in Figure 7a–d. According to the results, the 9-year-old boys had lower accuracy at the 15° and −45° angles and higher accuracy at the −15° and 45° angles, indicating that the −15° and 45°angles were more suitable for them. The accuracy normalization values of the girls at all four angles were above 0.8, indicating that the game performance of 9-year-old girls was significantly better than that of boys at the same age. Additionally, the results obtained at a 45° angle were significantly better than those obtained at other positions. The concentration of 9-year-old girls was significantly better than that of boys, and the concentration training results obtained in chair 4 were significantly better than those obtained in the other positions. As shown in the figure, the standard scores of 10-year-old children at all four angles were above 0.82, indicating that children in this age group had a better attention level, and the 45° angle was also the best angle for the game. The 11-year-old boys’ scores at the −15° angle were low, and the scores at all other angles were above 0.85, indicating that the game angles of −45°, 15°, and 45° were better. The accuracy normalization values of girls were generally above 0.8, indicating that girls had better concentration. The accuracy normalization values for 12-year-old children were above 0.8, and boys’ scores were generally lower than the girls’ scores, indicating that the four directions were more suitable for concentration training for children at this age, and children in this age group were less active than in the younger age group.

**Figure 8:**
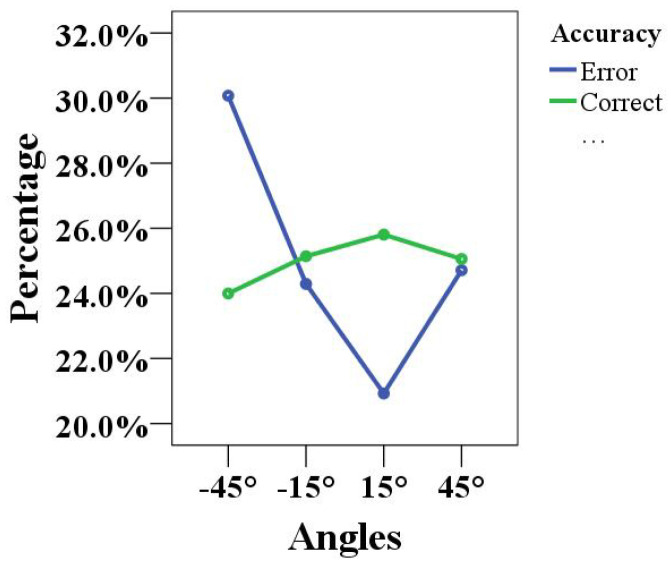
Analysis of the accuracy results obtained at different angles.

In addition, a summary analysis of the accuracy of the children’s completion of the game at four different angles (blue line indicates the error percentage, green line indicates the correct percentage) is shown in Figure 8. The error percentage at the −45° angle was close to 30.0%, and the accuracy rate was less than 25%, indicating that the concentration training at this angle was not ideal. The 45° angle exhibited the opposite result, with an accuracy rate higher than the error percentage. There was a great difference between the accuracy rate and the error percentage at the other two angles (−15° and 15°). Therefore, it was once again proven that the concentration training effect of the 45° angle in human–robot interaction games was the most significant.

**Figure 9:**
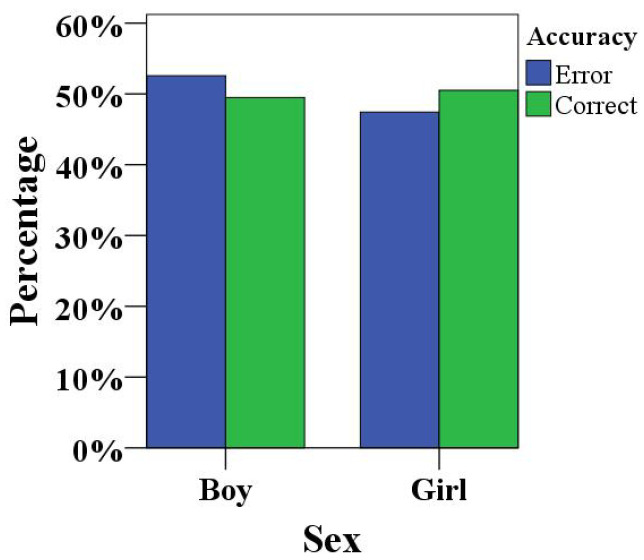
Analysis of the accuracy percentages for children of different sexes.

On the other hand, we have summarized the accuracy of results obtained by children participating in the HRI concentration training games, sorted by sex, as shown in Figure 9. The accuracy rate, in terms of concentration, in children of different sexes was distributed between 49% and 50%, and the error rate was distributed between 52% and 53%, which showed that the accuracy rate of the boys’ game performance was average. The accuracy rates of the girls’ game results were distributed between 50% and 51%, and the error rate was distributed between 47% and 48%, which was lower than the error rates of the boys’ game results, which showed that there were certain differences in the accuracy rates of children of different sexes in the HRI concentration training, but they were not significant.

**Figure 10:**
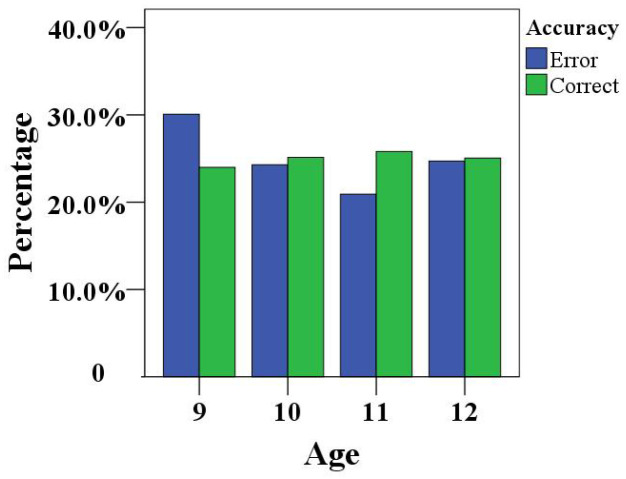
Distribution of grades across age groups.

According to the comprehensive analysis of the game performance of children of different ages, as shown in Figure 10, the error rate of the gesture completion error rate of 9-year-old children was slightly higher than the correct rate, whereas the accuracy rate among 11-year-old children was higher than the error rate, and the gaps between the accuracy rates and error rates obtained by 10-year-old and those obtained by 12-year-old children were very small. It can be seen that the concentration performance of 9-year-old children was poor; the performance of the other children were better.

### 4.3. Response Time

**Figure 11:**
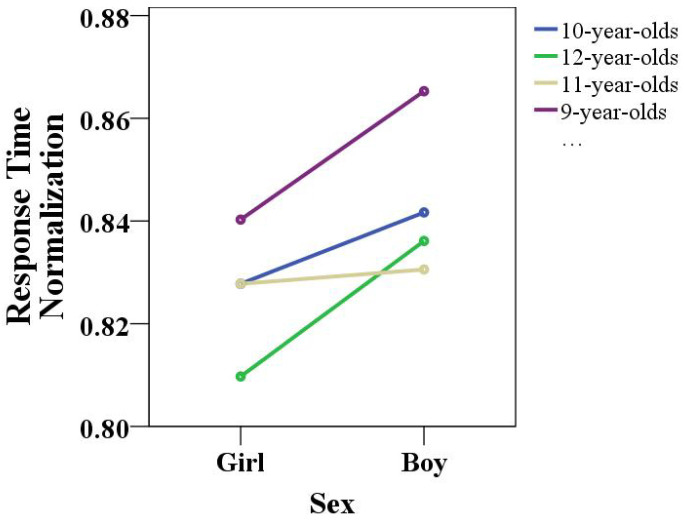
Comparison of response times for different ages and sexes.

The response time is an important indicator of children’s concentration traits. During the game, the game system recorded all children’s scores in real time; that is, the time difference from the robot issuing the gesture instructions to the child making the corresponding gesture feedback was recorded. There were certain differences in the reaction times of children of different ages. The average of the play response time for children of each age was normalized, and the results are shown in Figure 11 A normalized value of 0.8 indicated a response time of 1 s, and 0.88 indicated a response time of 3 s. During the experiment, the calculation time error due to the inherent performance of the equipment was about 0.4 s.

According to the comprehensive analysis, the 12-year-olds had the fastest reaction time, whereas the 9-year-olds had the slowest relative reaction time. The gap in reaction time between 10- and and 11-year-olds was small. In addition, a comprehensive analysis of the reaction times of children of different ages showed that the reaction time of girls was faster than that of boys. The response time was automatically calculated by the HRI game platform without human intervention, so the testing of this time interval was very simple, efficient, and reliable. Since the feedback time interval of children’s gestures is an important indicator of children’s concentration, testing the children’s feedback time provides an important basis for verifying the HRI game as an effective method for children’s concentration training. The difference in game reaction time proved that the HRI game can be used to carry out concentration training reliably, efficiently, and easily.

## 5. Conclusions

We proposed a concentration training method based on immersive HRI games. The immersive HRI game consisted of four functional modules, including a definition of the system’s geometry, the construction of a gesture training model, a deep learning algorithm, and providing feedback for the gesture recognition results. The deep learning algorithm YOLOv5 was used to realize gesture recognition, which ensured the real-time use and accuracy of the HRI gesture game. We built a gesture recognition model containing 10,000 pictures of children’s gestures, using the YOLOv5 algorithm to achieve a gesture recognition accuracy of 98.7%.

As an exploratory study, in order to verify the effectiveness of the HRI game in children’s concentration training, we specially invited 120 children aged from 9 to 12 to participate in the experiments. The factors that influenced the effect of concentration training, such as distance, direction, and age, were experimentally analyzed, which provided important experimental reference data for the subsequent design of the robot applied for HRI. We obtained 8640 experimental data. The aim of this experiment was to test the indicators of object recognition, visual short-term memory, and response time in order to evaluate children’s concentration. According to the analysis of the experimental results, we found that the subjects’ gender, age, distance, and orientaiton in relation to the camera all had a significant impact on the experimental results during the HRI game. The experimental results showed that a game distance of 1.5 m and an orientation of 45° were the best conditions for children to play concentration training games. Girls performed significantly better than boys. The influence of the effect of the position among the older children was significantly weaker than that observed among the younger children. Additionally, the response time of girls was lower than that of boys. All these results showed that immersive HRI games based on gesture recognition could provide a concentration training platform for children.

The immersive HRI game proposed in this paper was played in a preset game scenario without third-party interference between children and robots. Furthermore, the reliability, effectiveness, real-time performance, and convenience of the proposed method are verified. The theoretical basis is to further promote the application and development of HRI games in concentration training. As this paper is a preliminary study, the focus is on performance and game reaction times to demonstrate the feasibility of applying the method to attention assessment and training. Due to the limitations of experimental conditions and game content, the game method proposed in this paper has not fully verified all indicators of children’s attention training. Meanwhile, we don’t study the improvement effect of children’s concentration training by using the HRI game method proposed in this paper. However, the research results of this paper can provide a reference for the subsequent design of HRI games and educational robots. In the next phase, we will further optimize the content of the game to improve the effectiveness of concentration training.

## Figures and Tables

**Table 1:** Comparison between structures of YOLOv3, YOLOv4, and YOLOv5 [29].

	YOLOv3	YOLOv4	YOLOv5
Network Type	Full convolution	Full convolution	Full convolution
Backbone Feature	Darknet-53	CSPDarknet53	CSPDarknet53
Extractor			
Loss Function	Binary cross entropy	Binary cross entropy	Binary cross entropy
			Logits loss function
Neck	FPN	SSP and PANet	PANet
Head	YOLO layer	YOLO layer	YOLO layer

## Data Availability

The data presented in this study are available on request from the corresponding author. The data are not publicly available due to privacy reasons.

## References

[1] Yuan F., Klavon E., Liu Z., Lopez R.P., Zhao X. (2021). A Systematic Review of Robotic Rehabilitation for Cognitive Training. Front. Robot. AI.

[2] Liu W., Anguelov D., Erhan D., Szegedy C., Reed S.E., Fu C., Berg A.C., Leibe B., Matas J., Sebe N., Welling M. (2016). SSD: Single Shot MultiBox Detector. Proceedings of the Computer Vision—ECCV 2016—14th European Conference.

[3] Lowe D.G. (2004). Distinctive Image Features from Scale-Invariant Keypoints. Int. J. Comput. Vis..

[4] Dalal N., Triggs B. Histograms of Oriented Gradients for Human Detection. Proceedings of the 2005 IEEE Computer Society Conference on Computer Vision and Pattern Recognition (CVPR 2005).

[5] Krizhevsky A., Sutskever I., Hinton G.E., Bartlett P.L., Pereira F.C.N., Burges C.J.C., Bottou L., Weinberger K.Q. (2012). ImageNet Classification with Deep Convolutional Neural Networks. Proceedings of the Advances in Neural Information Processing Systems 25: 26th Annual Conference on Neural Information Processing Systems 2012.

[6] Deng J., Dong W., Socher R., Li L., Li K., Li F.-F. ImageNet: A large-scale hierarchical image database. Proceedings of the 2009 IEEE Computer Society Conference on Computer Vision and Pattern Recognition (CVPR 2009).

[7] Simonyan K., Zisserman A. Very Deep Convolutional Networks for Large-Scale Image Recognition. Proceedings of the 3rd International Conference on Learning Representations, ICLR 2015.

[8] Szegedy C., Liu W., Jia Y., Sermanet P., Reed S.E., Anguelov D., Erhan D., Vanhoucke V., Rabinovich A. Going deeper with convolutions. Proceedings of the IEEE Conference on Computer Vision and Pattern Recognition, CVPR 2015.

[9] Felzenszwalb P.F., Girshick R.B., McAllester D.A., Ramanan D. (2010). Object Detection with Discriminatively Trained Part-Based Models. IEEE Trans. Pattern Anal. Mach. Intell..

[10] Jiao L., Zhang F., Liu F., Yang S., Li L., Feng Z., Qu R. (2019). A Survey of Deep Learning-Based Object Detection. IEEE Access.

[11] Blackwell T., Bown O., Young M. (2012). Live Algorithms: Towards Autonomous Computer Improvisers. Computers and Creativity.

[12] Miskam M.A., Shamsuddin S., Yussof H., Ariffin I.M., Omar A.R. A questionnaire-based survey: Therapist’s response on emotions gestures using humanoid robot for autism. Proceedings of the 2015 International Symposium on Micro-NanoMechatronics and Human Science, MHS 2015.

[13] Alves-Oliveira P., Arriaga P., Paiva A., Hoffman G. YOLO, a Robot for Creativity: A Co-Design Study with Children. Proceedings of the 2017 Conference on Interaction Design and Children.

[14] Ali S., Moroso T., Breazeal C. Can Children Learn Creativity from a Social Robot?. Proceedings of the 2019 ACM SIGCHI Conference on Creativity and Cognition, C&C 2019.

[15] Chen H., Park H.W., Breazeal C. (2020). Teaching and learning with children: Impact of reciprocal peer learning with a social robot on children’s learning and emotive engagement. Comput. Educ..

[16] Cook A., Encarnação P., Adams K. (2010). Robots: Assistive technologies for play, learning and cognitive development. Technol. Disabil..

[17] Anzulewicz A., Sobota K., Delafield-Butt J. (2016). Toward the Autism Motor Signature: Gesture patterns during smart tablet gameplay identify children with autism. Sci. Rep..

[18] Akalin N., Uluer P., Kose-Bagci H., Ince G. Humanoid robots communication with participants using sign language: An interaction based sign language game. Proceedings of the 2013 IEEE Workshop on Advanced Robotics and Its Social Impacts.

[19] Tan M., Pang R., Le Q.V. EfficientDet: Scalable and Efficient Object Detection. Proceedings of the 2020 IEEE/CVF Conference on Computer Vision and Pattern Recognition, CVPR 2020.

[20] Bochkovskiy A., Wang C., Liao H.M. (2020). YOLOv4: Optimal Speed and Accuracy of Object Detection. arXiv.

[21] McNab F., Varrone A., Farde L., Jucaite A., Bystritsky P., Forssberg H., Klingberg T. (2009). Changes in Cortical Dopamine D1 Receptor Binding Associated with Cognitive Training. Science.

[22] Rahman M.A., Wang Y., Bebis G., Boyle R., Parvin B., Koracin D., Porikli F., Skaff S., Entezari A., Min J., Iwai D., Sadagic A. (2016). Optimizing Intersection-Over-Union in Deep Neural Networks for Image Segmentation. Proceedings of the Advances in Visual Computing—12th International Symposium, ISVC 2016.

[23] Rezatofighi H., Tsoi N., Gwak J., Sadeghian A., Reid I.D., Savarese S. Generalized Intersection Over Union: A Metric and a Loss for Bounding Box Regression. Proceedings of the IEEE Conference on Computer Vision and Pattern Recognition, CVPR 2019.

[24] Zörner S., Arts E., Vasiljevic B., Srivastava A., Schmalzl F., Mir G., Bhatia K., Strahl E., Peters A., Alpay T. (2021). An Immersive Investment Game to Study Human-Robot Trust. Front. Robot. AI.

[25] Logan D.E., Breazeal C., Goodwin M.S., Jeong S., O’Connell B., Smith-Freedman D., Heathers J., Weinstock P. (2019). Social Robots for Hospitalized Children. Pediatrics.

[26] Redmon J., Divvala S.K., Girshick R.B., Farhadi A. You Only Look Once: Unified, Real-Time Object Detection. Proceedings of the 2016 IEEE Conference on Computer Vision and Pattern Recognition, CVPR 2016.

[27] Redmon J., Farhadi A. (2018). YOLOv3: An Incremental Improvement. arXiv.

[28] Redmon J., Farhadi A. (2016). YOLO9000: Better, Faster, Stronger. arXiv.

[29] Nepal U., Eslamiat H. (2022). Comparing YOLOv3, YOLOv4 and YOLOv5 for Autonomous Landing Spot Detection in Faulty UAVs. Sensors.

[30] Bernotat J., Eyssel F., Sachse J. (2021). The (Fe)male Robot: How Robot Body Shape Impacts First Impressions and Trust Towards Robots. Int. J. Soc. Robot..

[31] Clarke A.R., Barry R.J., McCarthy R., Selikowitz M., Johnstone S.J. (2008). Effects of imipramine hydrochloride on the EEG of children with Attention-Deficit/Hyperactivity Disorder who are non-responsive to stimulants. Int. J. Psychophysiol..

[32] Baer R. (2015). Assessing Mindfulness in Multiple Contexts: A Comment on Christopher, Woodrich, and Tiernan (2014). Mindfulness.

[33] Arns M., Conners C.K., Kraemer H.C. (2013). A decade of EEG Theta/Beta Ratio Research in ADHD: A meta-analysis. J. Atten. Disord..

[34] Chen Y., Shen J. (2022). Core indicators of an evaluation and guidance system for quality of care in inflammatory bowel disease centers: A critical review. eClinicalMedicine.

[35] Xu Y., Jeong S. (2015). The contribution of human superior intra-parietal sulcus to visual short-term memory and perception. Mechanisms of Sensory Working Memory: Attention and Performance XXV.

[36] Mitchell D.J., Cusack R., Cam-CAN (2018). Visual short-term memory through the lifespan: Preserved benefits of context and metacognition. Psychol. Aging.

[37] Liu L., Liu Y., Gao X.Z. (2021). Impacts of human robot proxemics on human concentration-training games with humanoid robots. Healthcare.

